# Lithium toxicity. A case report

**DOI:** 10.1192/j.eurpsy.2021.1728

**Published:** 2021-08-13

**Authors:** E. Rodríguez Vázquez, C. Capella Meseguer, I. Santos Carrasco, J. Gonçalves Cerejeira, A. Gonzaga Ramírez, M. Queipo De Llano De La Viuda, G. Guerra Valera, C. De Andrés Lobo, T. Jiménez Aparicio, C. Vallecillo Adame

**Affiliations:** 1 Psiquiatría, Hospital Clínico Universitario de Valladolid, Valladolid, Spain; 2 Psiquiatría, HCUV, Valladolid, Spain; 3 Psiquiatría, Hospital Clínico Universitario Valladolid, Valladolid, Spain; 4 Psiquiatría, Hospital Clínico Universitario Valldolid, Valladolid, Spain

**Keywords:** lithium toxicity, bipolar disorder, lithium

## Abstract

**Introduction:**

Lithium is widely used in the treatment of the bipolar disorder. Once introduced, it is necessary to carry out an adequate control of the therapeutic range, since it is potentially toxic, and can affect various organs.

**Objectives:**

To present the case of a patient suffering from lithium poisoning and to review the symptoms of lithium poisoning.

**Methods:**

A descriptive study of a clinical case and review of the literature

**Results:**

49-year-old woman, married. Diagnosed with bipolar disorder. She went to the emergency room due to a low level of consciousness, kidney failure, trembling of the limbs, hyperthermia and leukocytosis. In the last two weeks, the patient has reduced her intake of food, not water, finding herself more and more shaky and less reactive. Lithium in blood at admission 1.71, so conventional dialysis was performed with a progressive decrease into 0.65. On examination, he is practically mutist, bradypsychia with a significant response latency. Clinical judgment: Accidental lithium poisoning.

**Conclusions:**

The primary site of toxicity is the central nervous system and clinical manifestations vary from asymptomatic supratherapeutic drug concentrations to clinical toxicity such as confusion, ataxia, or seizures. Severe lithium neurotoxicity occurs almost exclusively in the context of chronic therapeutic administration of lithium and rarely results from acute ingestion of lithium, even in patients currently taking lithium. As such it is an iatrogenic illness, occurring in patients who have identifiable clinical risk factors: nephrogenic diabetes insipidus, older age, abnormal thyroid function and impaired renal function.

**Disclosure:**

No significant relationships.

